# O-33 - LEARNING FROM DELIVERY: A QUALITATIVE EXPLORATION OF STAKEHOLDER PERSPECTIVES ON THE INFANT RSV IMMUNISATION PROGRAMME 2025/2026 IN IRELAND

**DOI:** 10.1093/pubmed/fdag046.029

**Published:** 2026-07-09

**Authors:** Dr Jane Finucane, Dr Kevin Brown, Dr Augustine Pereira, Dr Lois O'Connor, Dr Éamonn O'Moore

**Affiliations:** HSE Health Protection Surveillance Centre; Department of Public Health; National Health Protection Office, HSE; National Health Protection Office, HSE; National Health Protection Office, HSE

## Abstract

**Background:**

Respiratory Syncytial Virus (RSV) remains a leading cause of respiratory illness and hospitalisation in infants. Ireland successfully delivered an RSV immunisation programme through maternity based delivery during the 2024/2025 RSV season. For the 2025/2026 season, a community-based catch-up programme was added for infants born between March and August 2025, to run alongside a maternity service-based programme, for infants born 01/09/2025-28/02/2026. Capturing frontline stakeholder experience is essential to optimise delivery, equity and sustainability of this new programme.

**Objectives:**

To explore stakeholder perspectives on planning, communication, operational delivery, effectiveness, acceptability and equity of the 2025/2026 RSV immunisation programme, and to identify actionable lessons to inform future delivery.

**Methods:**

Virtual semi-structured focus groups were conducted with parents of immunised infants, community immunisation providers, regional Directors of Public Health, maternity Directors of Midwifery and general practitioners. Meetings were transcribed and analysed thematically.

**Results:**

Feedback on the community clinic experience from parents and providers was overwhelmingly positive, with high staff engagement in the-catch up programme. Across maternity services, delivery was smoother than the initial year, reflecting staff familiarity, improved logistics and refined workflows. The importance of year-round RSV education within antenatal care was consistently highlighted. Compressed timelines and limited lead-in for communications constrained preparedness for both maternity and community providers. Participants emphasised the need for wider national communications alongside targeted social media approaches, including engagement with trusted influencers. Equity challenges persisted, with lower uptake noticed in both maternity and community settings among migrant and socioeconomically disadvantaged families, partly mitigated through local outreach, text messaging, public health nurse engagement and targeted pop-up clinics.

**Conclusions:**

The 2025/2026 RSV immunisation programme demonstrated high acceptability and feasibility for providers and service users. Earlier national communication, assured funding, sustained year-round messaging and integrated equity-focused outreach are required to maximise impact, particularly for the catch-up programme.

